# Reproductive effects of cadmium on sperm function and early embryonic development *in vitro*

**DOI:** 10.1371/journal.pone.0186727

**Published:** 2017-11-02

**Authors:** Li-lin Zhao, Yan-fei Ru, Miao Liu, Jia-nan Tang, Ju-fen Zheng, Bin Wu, Yi-hua Gu, Hui-juan Shi

**Affiliations:** 1 Key Lab of Reproduction Regulation of NPFPC-Shanghai Institute of Planned Parenthood Research (SIPPR), Fudan University Reproduction and Development Institution, Shanghai, China; 2 Shanghai Key Laboratory of Reproductive Medicine, Shanghai Jiao Tong University, Shanghai, China; University of Missouri Columbia, UNITED STATES

## Abstract

Cadmium is a major environmental toxicant that is released into the atmosphere, water and soil in the form of cadmium oxide, cadmium chloride, or cadmium sulfide via industrial activities, such as the manufacturing of batteries and pigments, metal smelting and refining and municipal waste incineration. In the present study, we investigated the effects of cadmium exposure on sperm quality parameters, fertilization capacity and early embryonic development. Our study showed that *in vitro* incubation of human or mouse sperms with cadmium for a long time (up to 24 hours) could significantly decreased sperm motility in a concentration- and time-dependent manner. Exposure to cadmium in the environment for a short term (30 min) did not affect sperm motility but significantly reduced *in vitro* fertilization rate. We also evaluated the effects of cadmium at concentrations of 0.625 μg/ml, and 1.25 μg/ml on early embryonic development *in vitro* and observed that the blastocyst formation rate dramatically decreased with increasing cadmium concentration. This finding emphasizes the hazardous effects of cadmium on sperm quality as well as on natural embryo development and raises greater concerns regarding cadmium pollution.

## Introduction

Cadmium is one of the most toxic heavy metals, has no known beneficial biological function, and poses a significant public health hazard, including reproductive toxicity [1]. Cadmium is commonly used in various industrial products, such as nickel-cadmium batteries, computer components, pigments and glazes [2]. The general population may be exposed to cadmium through ingestion of contaminated food and drinking water, inhalation of particulates from ambient air, exposure to tobacco smoke, or ingestion of contaminated soil and dust [3]. Cadmium demonstrates a low excretion rate (biological half-life = 20–40 years), and accumulates mostly in liver, kidney and testes [4].

In recent years, ubiquitous cadmium pollution has drawn great concern due to its adverse effects on the reproductive system. A epidemiological study observed a negative association between seminal cadmium concentration and sperm concentration, sperm motility and percent abnormal spermatozoa [5]. Short-term intake of exposure to high doses of cadmium induced serious testicular injury (e.g., sterilization, necrosis, germ cell depletion, interstitial tissue damage and BTB (blood-testis barrier) disruption) in rodents [6, 7]. It has been demonstrated that low dosage of cadmium (50 μg/day, approximately 30- to 60-fold less than short-term doses) adversely affects mammalian reproductive function, with effects that include the disruption of testis and epididymis histology, damage to spermatogenesis, a decrease in sperm motility, a change in sperm morphology and a decrease in the acrosome reaction rate in rats [6, 7]. The epididymis and vas deferens are extremely important accessary organs that play vital roles in sperm maturation and storage. It has been demonstrated that cadmium exposure in rats (2 mg Cd/kg body mass/day) led to alkalization of the lumen fluid of the epididymis and vas deferens by direct inhibition of H-ATPase function [8]. Furthermore, the altered microenvironment damaged sperm function, including motility and capacitation. However, the current study investigating the adverse effects of cadmium on the male reproductive system focuses on tissue histology, epithelial cell function, sperm count, sperm morphology and male fertility, with very limited data from an assay of sperm function after ejaculation [9]. Despite a previous report demonstrating the adverse effects of direct cadmium exposure on spermatozoa *in vitro* [10], it remains unclear whether this paternal injury would result in defects in fertilization and a subsequent reduction in the developmental potential of embryos. Therefore, in this study we first evaluated the direct effect of cadmium upon sperm motility both in mice and humans. In addition, fertilization capacity as well as subsequent embryonic development are highly sensitive and reliable indicators to estimate sperm function. In vitro fertilization (IVF), which has become a clinical practice for infertility treatment, is also regarded as a sensitive screening system for reproductive toxicants [11]. A well-designed IVF assay can determine a single chemical’s toxicity at a specific stage of fertilization, and simplify understanding of the complicated physiological environment *in vivo*. Accordingly, in our study, we used IVF as an assay to estimate the fertilization capacity of mouse sperm previously exposed to cadmium in a short term and examined the early development of the resulting embryos. To better illustrate the development process of this type of early embryos, we also evaluated the effects of cadmium exposure on the *in-vitro* development of naturally occurred early embryo.

## Materials and methods

### Ethical statement

This study was carried out in full compliance with the Guide for the Care and Use of Laboratory Animals. The protocol was approved by the Committee on the Ethics of Animal Experiments of Shanghai Institute of Planned Parenthood Research. Specifically, normal fertile subjects were investigated at Shanghai JIAI Genetics & IVF Institute. The subjects provided their informed consent with a signature after receiving detailed explanations of the proposed study. The Ethics Committee of Shanghai Institute of Planned Parenthood Research approved all procedures.

### Animals

Approximately 6- to 8-week-old female B6D2F1 (C57BL/6×DBA/2, SIPPR-BK Animal Co., Ltd, Shanghai, China) strain mice were used as oocyte donors, and 10- to 15-week-old male B6D2F1 mice were used as sperm donors. A total of 18 male mice and 90 female mice were used in this study. All mice were housed under controlled light conditions (12 h light: 12 h dark) in the Laboratory Animal Services Facility and were fed a standard mouse diet and water ad libitum.

### Chemicals

Unless otherwise mentioned, chemicals were purchased from Sigma-Aldrich (St Louis, MO, USA). Cadmium acetate with a purity of 99.9% was dissolved at concentration of 1 mg/ml with double distilled water and stored at– 20°C as stock solution. Different concentrations of working solutions of cadmium acetate were made prior to use.

### Human and mouse sperm sample preparation

Fresh human semen samples were collected into sterile plastic containers by masturbation after 3–4 days of sexual abstinence and were then liquefied for 30 min at room temperature. Semen samples were then submitted to sperm washing followed by swim-up with modified human tubal fluid (mHTF, Millipore-Chemicon, Billerica, MA). Each washed sperm-mHTF was adjusted to 5–10×10^6^ cells/ml and divided into four groups randomly: 2.5 μg/ml, 5 μg/ml, and 10 μg/ml cadmium groups and a control group, by adding appropriate volumes of cadmium acetate stock solution.

Male mouse caudal epididymis was excised and dissected from the fat pad, blood vessels, and connective tissue. The caudal epididymis was transferred to glass dishes containing 1 ml BWW (Biggers, Whitten and Whittingham) medium pre-warmed to 37°C and cut into several places with iridectomy scissors to release spermatozoa into the medium. The BWW medium contains 25 mM NaHCO_3_ (Sigma-Aldrich), 1.7 mM CaCl_2_ (Sigma-Aldrich), 10 mM HEPES (Sigma-Aldrich) and 2.6% p/v bovine serum albumin (BSA; Sigma) [12]. In all cases, the pH was adjusted to 7.2–7.4. After 5 min, the sperm suspension was transferred to 5 ml centrifuge tubes. The concentration of sperm was adjusted to 3–4×10^6^ cells/ml by adding appropriate cadmium acetate stock solution. Next, the spermatozoa were subjected to capacitation by incubation at 37°C under 5% CO_2_ in air for various periods of time.

### Measurement of human and mouse sperm motility parameters

Human sperm samples (1 ml) were assigned to four groups (0 μg/ml, 2.5 μg/ml, 5 μg/ml and 10 μg/ml). The four groups of human sperm were incubated at 37°C for 24 h. Five time points within 24 hours (0 hours, 6 hours, 16 hours, 20 hours and 24 hours) were selected, and the sperm motility parameters for each sample were analyzed using a computer-assisted semen analysis (CASA) machine (HTM-TOX IVOS sperm motility analyzer, Animal Motility, version 12.3A; Hamilton Thorne Research). The instrument settings used during the analysis were: temperature, 37°C; minimum cell size, 2 pixels; minimum contrast, 35; minimum static contrast, 25; low average path velocity cutoff, 20.0; low straight-line velocity cutoff, 30.0; threshold straightness, 50%; static head size, 1.0–2.90; static head intensity, 0.6–1.4; and magnification, 1.89. Thirty frames were acquired at a frame rate of 60 Hz.

Mouse sperm suspensions (200 μl) were incubated with different concentrations of cadmium (0 μg/ml, 2.5 μg/ml, 5 μg/ml, and 10 μg/ml) in a CO_2_ incubator (5% CO_2_ in air at 37°C). Sperm aliquots incubated for various periods of time under different experimental conditions were placed in a Sperm Analysis Chamber (Hamilton Thorne Research, Beverly, USA) and analyzed using the computer-assisted semen analysis (CASA) machine (HTM-TOX IVOS sperm motility analyzer, Animal Motility, version 12.3A; Hamilton Thorne Research). The instrument settings used during analysis were: temperature, 37°C; minimum cell size, five pixels; minimum contrast, 50; minimum static contrast, 25; low average path velocity cutoff, 20.0; low straight-line velocity cutoff, 30.0; threshold straightness, 50%; static head size, 0.3–1.95; static head intensity, 0.5–1.3; and magnification, 0.89. Thirty frames were acquired at a frame rate of 60 Hz.

### Collection of mouse oocytes and zygotes

Mature female mice were super-ovulated first with 10 IU of equine chorionic gonadotropin (eCG) and after 48 h with 5 IU of human chorionic gonadotropin (HCG). Approximately 13–16 h after HCG administration, the female mice were killed by CO_2_ asphyxiation. Next, the cumulus oocyte complexes (COCs) were collected from the removed oviducts and then maintained in HTF medium supplemented with bovine serum albumin (HTF-BSA, Millipore-Chemicon, Billerica, MA) medium at 37°C in an atmosphere of 5% CO_2_.

With regard to the recovery of the naturally fertilized zygotes, female mice were mated with males and examined 12–18 h after HCG injection for the presence of copulation plugs. The female mice were killed by CO_2_ asphyxiation and fertilized oocytes were recovered by removed the oviducts 20 h after the HCG injection. The cumulus of zygotes was dispersed in 0.1% hyaluronidase (Sigma-Aldrich) and washed in several changes of Hepes-buffered CZB medium (HCZB). Fertilized oocytes (identified by the presence of a second polar body and two pro-nuclei) were then placed in potassium-chloride-supplemented simplex optimized medium (KSOM medium, Millipore-Chemicon, Billerica, MA), which was previously equilibrated in a humidified atmosphere of 5% CO_2_ in air at 37°C.

### *In vitro* fertilization (IVF) and subsequent embryonic development procedure

To investigate the effect of cadmium on fertilization and subsequent embryonic development, described in Chemicals section, stock solution of cadmium was used as a medium supplement. According to our preliminary experiments, we adopted three concentrations of 2.5 μg/ml, 5 μg/ml, and 10 μg/ml in the sperm exposure experiment both in mouse and human. Furthermore, we observed that cadmium exposure at a dose of 10 μg/ml for 30 minutes did not impair the motility of mouse spermatozoa. We placed mouse sperm, which were squeezed out of cauda epididymis, in cadmium-containing HTF-BSA for 30 min and then washed them with HTF-BSA before analyzing the motility parameters by CASA. Next, the sperm were incubated in fresh HTF-BSA medium for another 60 min until capacitation was complete, after which a normal IVF procedure was performed. HTF-BSA medium was equilibrated in a 37°C, 5% CO_2_ incubator one day before the experiment. A small volume of capacitated sperm suspension was added to a drop of 100 μl HTF-BSA medium containing freshly ovulated oocytes to achieve a final sperm concentration of 10^6^/ml. Four to six hours later, fertilized oocytes at the pronuclear stage were washed and cultured in KSOM for *in vitro* development to the morula/blastocyst stages in 5% CO_2_ in air. Oocytes were observed for male and female pronucleus formation (fertilization) at 6 h after the initiation of culture, and the numbers of 2-cell embryos, 4-cell embryos, morula and blastocysts after 24 h, 48 h, 72 h and 96 h in culture were checked and recorded, respectively.

### *In vitro* culture of embryos derived from natural fertilization

To study the post-fertilization effects of Cd on the development of embryos derived from natural fertilization, embryos were continuously exposed to cadmium from the zygote stage with 2 pronuclei produced by natural insemination to the blastocyst formation stage at exposure concentrations of 0.625 μg/ml or 1.25 μg/ml in KSOM culture medium *in vitro*. KSOM medium was equilibrated in a 37°C, 5% CO_2_ incubator one day before the experiment. Zygotes were observed and recorded at the initiation of culture, and the number of 2-cell embryos, 4-cell embryos, morulae and blastocysts after 12 h, 36 h, 60 h and 84 h, respectively, were checked and recorded.

### Statistical analysis

All data in this study were obtained from five independent experiments. The Shapiro-Wilk test was used to determine whether the data were normally distributed. If the data exhibited a normal distribution, the statistical comparisons of the mean differences between groups were performed using one-way analysis of variance (ANOVA) followed by Dunnett’s multiple comparisons test. If the data did not exhibit a normal distribution, the mean differences between groups were calculated using non-parametric tests. GraphPad 7.0 (GraphPad Software, USA) was used for the statistical analyses. The threshold for a statistically significant difference was set at a P-value of ≤0.05. Data are expressed as the mean ± the standard error of the mean (SEM).

## Results

### Cd^2+^ reduces sperm total motility and progressive motility

**Fig 1:**
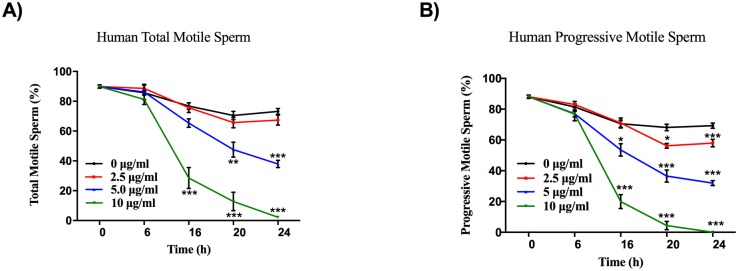
Cd^2+^ reduces human sperm total motility and progressive motility. Human sperms were treated with a series (0 μg/ml, 2.5 μg/ml, 5 μg/ml, 10 μg/ml) of Cd^2+^ concentrations for 6 h, 16 h, 20 h and 24 h, and the total motility (A) and progressive motility (B) of sperm were evaluated. Data represent the mean ± SEM, n = 5.

**Fig 2:**
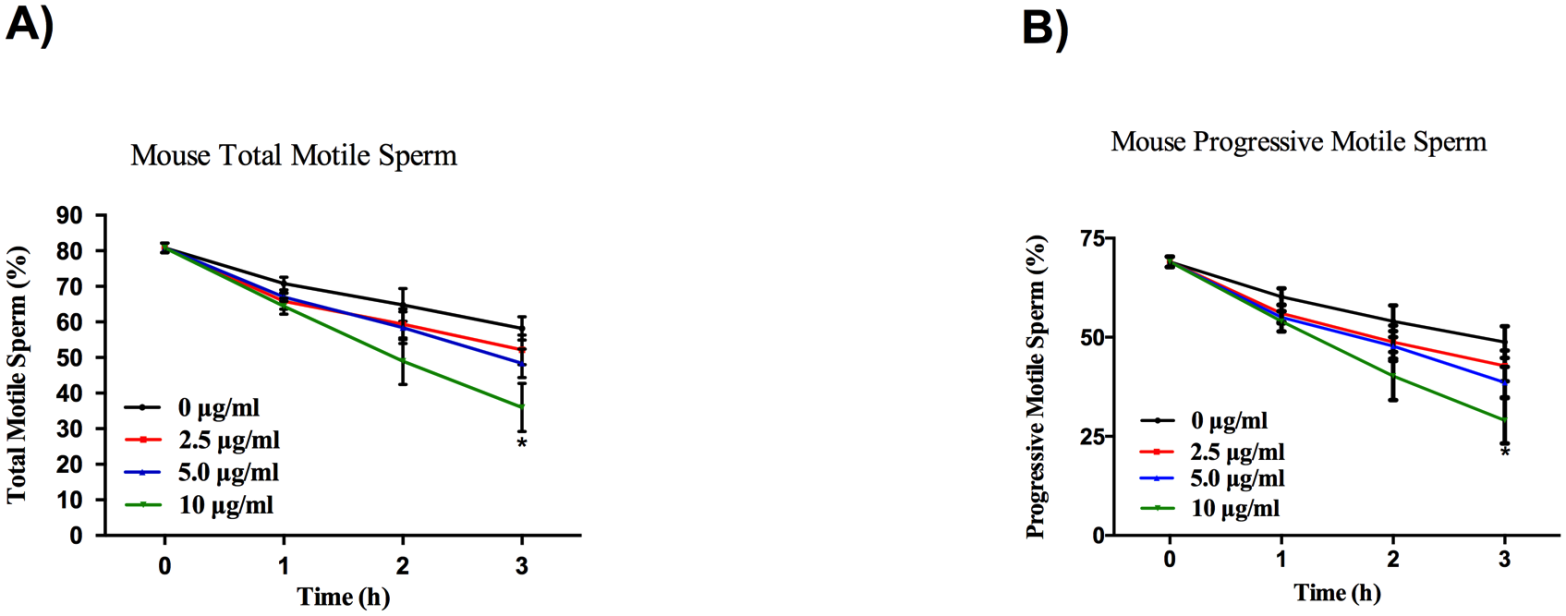
Cd^2+^ reduces mouse sperm total motility and progressive motility. Mouse sperm were treated with different concentrations (0 μg/ml, 2.5 μg/ml, 5 μg/ml, 10 μg/ml) of Cd^2+^ for 1 h, 2 h, and 3 h, and the total motility (A) and progressive motility (B) of sperm were assessed. Data represent the mean ± SEM, n = 5.

With CASA, we objectively investigated the direct effects of cadmium on sperm parameters. As shown in Figs 1 and 2, Cd^2+^ led to a significant reduction in the percentage of total motile and progressively motile sperm in a dose- and time-dependent manner, both in human and mouse.

In detail, the total motility and progressive motility rates of human sperm exposed to 2.5 μg/ml, 5 μg/ml, and 10 μg/ml Cd^2+^ for 6 h were 88.64±2.15%, 86.24±5.21%, 81.18±3.32%, and 83.04±2.04%, 77.08±4.63%, 76.80±1.83%, respectively, compared to control values of 85.7±1.65% and 81.44±1.98%, respectively. The total motility and progressive motility rates of human sperm exposed to 2.5 μg/ml, 5 μg/ml, and 10 μg/ml Cd^2+^ for 16 h were 75.72 ±3.23%, 65.40±2.87%, 28.58±6.99% (P<0.001) and 71.02±3.29%, 53.54±4.03% (P<0.05), 19.98±4.53% (P<0.001), respectively, compared to control values of 76.76±2.21% and 70.5±2.31%, respectively. The total motility and progressive motility rates of human sperm exposed to 2.5 μg/ml, 5 μg/ml, 10 μg/ml Cd^2+^ for 20 h were 65.72±3.54%, 47.54±4.98% (P<0.01), 12.82±6.16% (P<0.001) and 56.22±1.47% (P<0.05), 36.6±3.95% (P<0.001), 4.34±2.69% (P<0.001), respectively, compared to control values of 70.42±2.81% and 68.14±2.08%, respectively. The total motility and progressive motility rates of human sperm exposed to 2.5 μg/ml, 5 μg/ml, 10 μg/ml Cd^2+^ for 24 h were 67.40±3.37%, 37.98±2.47% (P<0.001), 2.20±0.90% (P<0.001) and 57.94±2.46% (P<0.001), 31.92±1.63% (P<0.001), 0% (P<0.001), respectively, compared to control values of 73.22±1.92% and 69.26±1.71%, respectively (Fig 1).

As for mouse sperm motility, the total motility and progressive motility rates of mouse sperm exposed to 2.5 μg/ml, 5 μg/ml, 10 μg/ml Cd^2+^ for 1 h were 65.80±2.27%, 67.00±1.23%, 64.40±2.23% and 56.00±2.24%, 55.00±1.52%, 54.00±2.57%, respectively, compared to control values of 70.80±1.77% and 60.20±2.15%, respectively. The total motility and progressive motility rates of mouse sperm exposed to 2.5 μg/ml, 5 μg/ml, 10 μg/ml Cd^2+^ for 2 h were 59.40±4.31%, 58.40±4.48%, 49.00±6.54% and 48.80±4.20%, 47.80±3.75%, 40.20±6.11%, respectively, compared to control values of 64.80±4.64% and 54.00±4.00%, respectively. The total motility and progressive motility rates of mouse sperm exposed to 2.5 μg/ml, 5 μg/ml, 10 μg/ml of Cd^2+^ for 3 h were 52.20±4.16%, 48.40±3.99%, 36.00±6.80% (P<0.01) and 42.80±3.89%, 38.60±3.95%,29.00±5.86% (P<0.05), respectively, compared to control values of 58.20±3.32% and 48.80±3.99%, respectively (Fig 2).

### Influences of cadmium exposure on sperm fertilization and subsequent embryonic development *in vitro*

Following exposure to Cd using HTF medium containing different concentrations (2.5 μg/ml, 5 μg/ml and 10 μg/ml) of Cd^2+^ for 30 min, sperm were washed in HTF, and the quality parameters were analyzed by CASA. Data revealed that the total motility and progressive motility of spermatozoa showed no obvious difference from those of controls when spermatozoa were incubated in HTF-cadmium (0 μg/ml, 2.5 μg/ml, 5 μg/ml and 10 μg/ml) for 30 min. Next, spermatozoa in the four groups were adjusted to a concentration of 1×10^6^/ml and then used in an IVF procedure. Fertilized oocytes were judged normal by extrusion of the second polar body and the presence of 2 pronuclei, which indicate successful fertilization. As shown in Table 1, all four groups of spermatozoa retained their fertilization potential; however, the rate of pronuclei formation (fertilization) was significantly reduced in a dose- and time-dependent manner compared to that in the control group. However, the first cleavage, 4-cell/8-cell, morula and blastocyst formation rates in the groups treated with different Cd^2+^ concentrations exhibited no significant difference compared with the values observed in the non-treated control group (Table 1).

**Table 1:** Effects of cadmium on mouse sperm fertilization capability in the IVF procedure and on subsequent embryonic development.

Category	No. of oocytes (replicates)	No. with pronuclei formation (%)^a^	No. of 2-cell embryos (%)^b^	No. of 4-cell embryos (%)^b^	No. of 8-cell embryos (%)^b^	No. of morulae (%)^b^	No. of blastocysts (%)^b^
**Control**	199 (5)	186 (92.8)	181 (96.6)	176 (94.7)	176 (94.7)	176 (94.7)	159 (86.8)
**2.5μg/ml**	179 (5)	131 (71.9)**	129 (98.3)	124 (94.8)	124 (94.8)	122 (93.2)	117 (88.4)
**5μg/ml**	214 (5)	121 (53.9)***^,^^##^	115 (95.8)	115 (95.8)	115 (95.8)	114 (95.1)	108 (88.6)
**10μg/ml**	113 (5)	15 (13.3)***^,^^###^^,^^$ $ $^	15 (100.0)	14 (97.1)	14 (97.1)	14 (94.1)	12 (84.3)

^a^Based on total oocytes.

^b^Based on total pronuclear embryos.

**, ^##^p<0.01,

***, ^###, $ $ $^p<0.001, comparisons were made between the control group and each treated group, between the 2.5 μg/ml group and the other two concentration groups, and between the 5 μg/ml and 10 μg/ml concentration groups, respectively.

### Influences of cadmium exposure on the development of embryos derived from natural fertilization *in vitro*

Considering that the potential hazards of toxicants has been suggested to vary according to the different developmental and differentiation states of target cells and tissues [13], and since the detrimental effect of low-level Cd^2+^ direct exposure on spermatozoa motility and fertilization had been observed, we were further concerned about whether naturally fertilized oocytes were influenced by Cd^2+^. An *in vitro* culture experiment was set up in which embryos derived from natural insemination were divided into three groups at random: Cd^2+^-free KSOM, 0.625 μg/ml Cd^2+^-added KSOM, and 1.25 μg/ml Cd^2+^-added KSOM. As shown in Table 2, the 0.625 μg/ml Cd^2+^-added group exhibited a significant decline in the percentage of 2-cell embryos, 4-cell/8-cell embryos, morulae and blastocysts compared to the Cd^2+^-free group (P < 0.001). However, the 1.25 μg/ml Cd^2+^-added medium had a dramatic impact on 2-cell embryo formation, causing 2-cell arrest (Table 2).

**Table 2:** Effects of cadmium on the development of naturally fertilized mouse zygotes.

Category	No. of oocytes with pronuclei (replicates)	No. of 2-cell embryos (%)	No. of 4-cell embryos (%)	No. of 8-cell embryos (%)	No. of morulae (%)	No. of blastocysts (%)
**Control**	167 (5)	158 (93.2)	158 (93.2)	157 (92.2)	157 (92.2)	144 (83.6)
**0.625μg/ml**	222 (5)	184 (82.0) *	171 (76.4) ***	170 (75.4) ***	170 (75.4) ***	99 (46.8) ***
**1.25μg/ml**	266 (5)	213 (78.2) **	0 (0.0)	0 (0.0)	0(0.0)	0 (0.0)

Based on total oocytes with pronuclei.

***p < 0.001, comparisons were made between the control group and each treated group and between the 0.625 μg/ml and 1.25 μg/ml concentration groups, respectively.

## Discussion

Cadmium has been demonstrated to impair mammalian reproduction in recent studies. Destruction of testis caused by Cd^2+^ has been carefully studied [14–17]. However, the sperm are another key cell type affected by Cd^2+^, and given their critical role in male reproduction, the deleterious effects of Cd^2+^ exposure are of great clinical importance. In the present study, we used a set of promising *in vitro* models for reproductive toxicology [18] and examined the direct effects of Cd^2+^ on spermatozoa, fertilization and early embryo development, extrapolating experimental data that demonstrated the human reproductive hazard posed by Cd^2+^. Our findings revealed that Cd^2+^ reduced the motility of sperm *in vitro* and affected the penetration of sperm into oocytes and the development of embryos derived from natural fertilization.

Several studies have already reported diminution of sperm motility in individuals exposed to Cd^2+^, including smokers [19]. In our study, the results demonstrate that Cd^2+^ dramatically decreased the percentage of total motile and progressively motile sperm in a dose- and time-dependent manner, not only in humans but also in mice (Figs 1 and 2). In line with our opinion that Cd^2+^ may reduce the motility of human spermatozoa, a previous study analyzing the effect of Cd^2+^ on the motility and vitality of human spermatozoa *in vitro* indicated that Cd^2+^ significantly diminished the progressive motility of human spermatozoa within their concentration range without affecting sperm vitality. Notably, the Cd^2+^ concentration used in the present study was higher than that used in the abovementioned study. Given the different criteria for screening human sperm samples, one possible explanation for this discrepancy is that the quality of sperm used in the present study was higher than that employed by Da, C.R., et al. [20]. Consistent with our notion that Cd^2+^ may reduce the motility of mouse spermatozoa, a study adopted a series of Cd^2+^ concentrations (0.1 μM, 0.5 μM, 1 μM, 5 μM, 10 μM, 50 μM) *in vitro* and a similar injection dosage (1.2 mg/kg BW) *in vivo*, and demonstrated that Cd^2+^ promoted tyrosine phosphorylation of dihydrolipoamide dehydrogenase (DLD), inhibited its dehydrogenase activity, and thus decreased adenosine triphosphate (ATP) production and sperm motility. However, the abovementioned study focuses only on the mechanism of action of cadmium on sperm motility and does not demonstrate a dose- and time-dependent effect of cadmium on sperm motility [21].

Furthermore, the range of environmental Cd exposure levels is very broad and varies according to the evaluation method, survey object and region. According to the latest literature report, environmental cadmium exposure levels were observed as follows: blood Cd concentrations from 0 μg/l to 5.0 μg/l, urine Cd concentrations from 0 μg/l to 9.8 μg/l and semen Cd concentrations from 0 μg/l to 5.92 μg/l [5, 22]. Moreover, the biological half-life of Cd was too long, and urinary Cd concentrations kept increasing with age [23], indicating that Cd exposure exerts a cumulative effect on organisms with long-term exposure. Our data from the cadmium exposure mouse model demonstrated that the testis Cd concentration is approximately twice the blood Cd concentration; therefore, the blood Cd level did not perfectly reflect the actual situation in the male reproductive system. Environmental exposure is an extremely complicated process, and Cadmium exposure is often accompanied by exposure to lead and magnesium [5]. Therefore, a combination effect is common in humans with exposure to cadmium. However, for a single-factor *in vitro* study, it is helpful to use higher concentrations than those used in *in vivo* studies. Furthermore, the higher concentration of cadmium is closer to occupational and acute exposure. Accordingly, in this study, we used a much higher Cd concentration than that reported in the existing literature reports. Nevertheless, we intend to refine the selection of Cd concentration for future study.

For decades, an increasing number of infertile couples have resorted to assisted reproduction technology (ART) to conceive a child. Therefore, it is of great importance to clarify the effect of Cd exposure on in vitro fertilization (IVF). According to the literature on exposure, there are three research methods and perspectives regarding the effect of Cd on IVF: 1. Oocyte exposure (maternal effect). It has been well-documented that Cd exposure negatively affects oocyte maturation, subsequent fertilization and embryo development [24]. 2. Sperm and oocyte exposure in culture media (parental effect). No reports were found about this type of exposure. 3. Sperm exposure (paternal effect). Keewan Kim et al. suggest an inverse adjusted association between blood cadmium in men and oocyte fertilization [25]. However, the causality between sperm Cd exposure and IVF outcomes is far from settled; therefore, we focused on this part in consideration of the purpose of this study. Our experimental results demonstrated that incubation of sperm in cadmium-added HTF medium for short time (30 min) to implement IVF did not significantly alter the total motility and progressive motilty of spermatozoa compared to that observed in control spermatozoa (Fig 2). Upon induction of the IVF process, differences among the spermatozoa caused by pretreatment with different concentrations Cd^2+^ could still be detected in the processes of fertilization and subsequent embryonic development. The IVF procedure includes sperm-egg binding, zygote formation and the first cleavage to form a 2-cell embryo. After transfer of the zygote into KSOM medium, the pronucleus undergoes multiple cleavages to form a 2-cell embryo, 4-cell embryo, morula then blastocyst successively in vitro. Although Cd^2+^ exposure reduces the fertilization rate with increasing Cd^2+^ concentration, oocytes fertilized with these spermatozoa still retained their full-term developmental potential after being transferred (Table 1). This fact confirmed the hypothesis that exposure to Cd^2+^ from various sources affects sperm quality. However, although sperm fertilization ability was diminished, Cd^2+^-contaminated sperm did not affect the process of early embryo development in our study, and it could be speculated that while infertile males suffer less serious contamination, IVF still serves as a sensible and feasible approach in ART. Unlike several other studies that detected toxic effects of some substances, for example, di-(2-ethylhexyl)-phthalate, acetamiprid, imidacloprid and nicotine, on sperm function and fertilization ability, our study demonstrated that toxic substances affect sperm function in addition to reducing the blastocyst formation rate [26, 27]. This difference may be because different pollutants do not have the same mechanism of action on sperm and embryo development. Moreover, it is necessary to evaluate the developmental potential and security of the blastocyst by implantation experiments in future studies.

To determine the impact of Cd^2+^ on early embryonic development, naturally fertilized zygotes were continuously exposed to Cd^2+^ at different concentrations (0 μg/ml, 0.625 μg/ml, and 1.25 μg/ml) until blastocyst formation. In the presence of the higher concentration (1.25 μg/ml) of Cd^2+^ in the culture medium, the first cleavage stage of naturally fertilized oocytes to form 2-cell embryos was remarkably inhibited, and all such embryos were arrested at the 2-cell stage. Although the lower concentration (0.625 μg/ml) of Cd^2+^ exposure in naturally fertilized oocytes affected the process of development from the first cleavage stage to the blastocyst stage, some embryos still retained full-term developmental potential (Table 2). The toxicity of cadmium to naturally fertilized oocytes is much higher than that observed for spermatozoa function in our results. Therefore, we infer that maternal cadmium exposure has a more severe adverse impact on the fetus than does paternal exposure.

The mechanisms underlying the hazardous effects of Cd^2+^ remain unclear. Oxidative stress, apoptosis, inflammation, and effect of cadmium on competitive suppression of zinc are possibly involved in these mechanisms [28–32]. Previous studies indicated that acute or chronic exposure to Cd^2+^ causes oxidative stress in animals and humans [32, 33], which could harm reproductive organs. Furthermore, Cd^2+^ generates reactive oxygen species (ROS) that induce apoptosis in testicular germ cells. A potential underlying signaling pathway may be activated through Cd-induced ROS and may be responsible for the upregulation of poly (ADP-ribose) polymerase-1 (PARP-1), translocation of apoptosis inducing factor (AIF) to the nucleus, and apoptosis of testicular cells in rat testes [34]. Some studies have demonstrated that the injury due to acute Cd^2+^ exposure activates a large number of inflammatory and cytotoxic mediators, especially the production of TNF-α and IL-1β [35]. Studies have also demonstrated that Cd^2+^ induces inflammation in the mouse testis [36], which may decrease the sperm parameters. Studies have demonstrated a preventive effect of Zn^2+^ against Cd^2+^-induced oxidative stress in the rat testis [30]. A competitive mechanism of interaction is a plausible mechanism for the protective effect of Zn^2+^ in relation to Cd^2+^ toxicity. Radiolabeled Zn has been reported to be incorporated into elongated spermatids and to display competitive interaction with heavy metals for incorporation [37]. Because Zn is an essential component of the oxidant defense system and functions at several levels [38] and Zn deficiency in the diet paves the way for cell damage in the rat testis [39], the competitive suppression effect of cadmium on zinc may lead to a decrease in sperm quality.

Taken together, the results of the present study unveiled the hazardous effects of direct exposure to Cd^2+^ on sperm quality, sperm penetration into oocytes, and the development of embryos derived from natural fertilization. The results indicated that direct exposure to Cd^2+^ exerted harmful effects on sperm function and embryonic development. These reliable data elucidated the reproductive toxicity of Cd^2+^ in mammals from a new perspective, i.e., the direct effects of Cd^2+^ on gametes, fertilization and embryonic development. Therefore, considering the current levels of cadmium pollution in some areas and the increased incidence of infertility in the population, our study to some extent broadens the knowledge about the harmful effects of heavy metals and underlines the great importance of preventing cadmium pollution.
